# Inward-facing conformation of l-ascorbate transporter suggests an elevator mechanism

**DOI:** 10.1038/s41421-018-0037-y

**Published:** 2018-07-17

**Authors:** Ping Luo, Shuliu Dai, Jianwei Zeng, Jinsong Duan, Hui Shi, Jiawei Wang

**Affiliations:** State Key Laboratory of Membrane Biology, Beijing Advanced Innovation Center for Structural Biology, School of Life Sciences, Tsinghua University, 100084 Beijing, China

## Abstract

Various bacteria can ferment vitamin C (l-ascorbate) under anaerobic conditions via the phosphoenolpyruvate-dependent phosphotransferase system (PTS). The PTS^asc^ system is composed of two soluble energy-coupling proteins (EI and HPr) and an enzyme II complex (EIIA, EIIB, and EIIC) for the anaerobic uptake of ascorbate and its phosphorylation to l-ascorbate 6-phosphate in vivo. Crystal structures of the ascorbate-bound EIIC component from *Escherichia coli* are available in outward-open and occluded conformations, suggesting a possible elevator mechanism of membrane transport. Despite these advances, it remains unclear how EIIC actually transports the substrate across the membrane and interacts with EIIB, which transfers its phosphate group to the EIIC-embedding ascorbate. Here, we present the crystal structure of the EIIC^asc^ component from *Pasteurella multocida* in the inward-facing conformation. By comparing three conformational states, we confirmed the original proposed model: the ascorbate translocation can be achieved by a rigid-body movement of the substrate-binding core domain relative to the V motif domain, which brings along the transmembrane helices TM2 and TM7 of the V motif domain to undergo a winding at the pivotal positions. Together with an in vivo transport assay, we completed the picture of the transport cycle of the ascorbate superfamily of membrane-spanning EIIC components of the PTS system.

## Introduction

The phosphoenolpyruvate (PEP)-dependent sugar phosphotransferase system (PTS), which catalyzes PEP-dependent group translocation, is used by bacteria for sugar uptake where the source of energy is from phosphoenolpyruvate^1,2^. The PTS includes an integral membrane transporter protein IIC, an enzyme IIB-like protein and an enzyme IIA-like protein forming an enzyme II (EII) complex, each of which is sugar specific^3^. All three proteins as well as the energy-coupling PTS proteins that are not specific for the transported sugar, enzyme I (EI) and the histidine phosphocarrier protein (HPr), are required for sugar uptake and its phosphorylation in vivo^1,3–5^. The EII components of the PTS can also fuse to each other forming multifunctional polypeptides made up of two or more domains^5^. The *Escherichia coli* K12 chromosome encodes 21 different PTS carbohydrate transporters^3^. The unusual mechanism of PTS-mediated sugar uptake tightly couples sugar transport to sugar phosphorylation in a “group translocation” process^6^. In the first step, EI of the PTS is autophosphorylated with a phosphate group originating from phosphoenolpyruvate. Then the phosphate group is transferred sequentially from EI to HPr, to EIIA, and to EIIB. Finally, EIIB transfers its phosphate group to the transported sugar bound to the membrane-spanning EIIC, and the phosphorylated sugar is then released into the cytoplasm^3,5,7^. The bacterial PTSs are classified into four evolutionary distinct (super) families according to the TCDB website (http://www.tcdb.org/superfamily.php)^3,6,8^: (i) the glucose/fructose/lactose (GFL) superfamily, which is the largest one and is subdivided into four families (TC# 4.A.1–4.A.4), (ii) the ascorbate/galactitol (AG) superfamily, which consists of two families (TC# 4.A.5 and 4.A.7), (iii) the mannose family (TC# 4.A.6), and (iv) the non-transporting dihydroxyacetone family.

In earlier reports, three crystal structures and one 2D electron crystallographic projection structure of PTS transporters had been solved: the ChbC diacetylchitobiose group translocator in the inward-facing occluded conformation^9^ and maltose transporter MalT in the outward-facing occluded conformation^10^ of the GFL superfamily, and the UlaA l-ascorbate group translocator in both outward-facing and occluded conformations^11^ of the AG superfamily and the glucose-specific EIIC transport domain of the GFL superfamily from *E. coli* solved by electron crystallography of tubular crystals in an inward-facing conformation^12^. EIIC proteins are thought to use the elevator mechanism^13–16^, an implementation of the alternate access mechanism^17^, to transport sugars across the membrane^18^. However, there has been no inward-facing 3D structure of EIIC in the AG superfamily available to validate the proposed elevator mechanism.

**Fig. 1:**
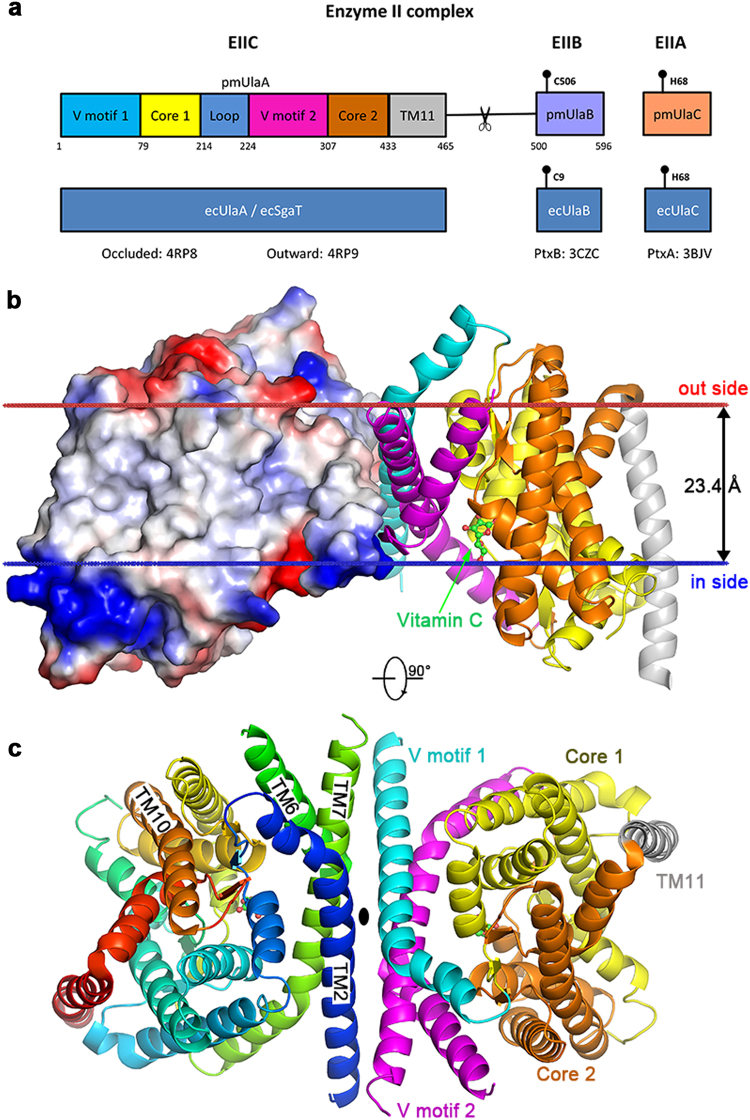
Domain composition of l-ascorbate PTS enzyme II complex and overall structure of pmUlaA. **a** In the wild type PTS EII complex from *Pasteurella multocida*, pmUlaB is fused C-terminal to pmUlaA to form a single peptide. His68 of UlaC accepts the phosphate group transferred from PEP, EI, HPr sequentially, and then gives it to Cys506/Cys9 of UlaB. Ultimately, UlaB transfers its phosphate group to the l-ascorbate. Residue 475, indicated by the scissor, was the proteolytic site with trypsin. **b** The structure of the pmUlaA homodimer is shown in cartoon and electron potential surface representations as viewed within the plane of the membrane. pmUlaA is spatially organized into V motif 1 (cyan), core 1 (yellow), V motif 2 (magenta), core 2 (orange) and TM11 (gray) subdomains. Vitamin C is shown in ball-and-stick representation. The spatial position of pmUlaA inside the lipid bilayer is predicted by the PPM server^24^. **c** View from the extracellular side of the pmUlaA homodimer. One protomer is shown in rainbow colors

Vitamin C (or l-ascorbic acid, or simply ascorbate) can be fermented by enteric bacteria, e.g., *E. coli*, via a PTS-AG system^19^. Two divergently transcribed operons, *ulaABCDEF/sgaTBAHUE* and *ulaG* located upstream of the *ula* operon (previously designated the *sga* operon), encode catabolic enzymes for the utilization of l-ascorbate, allowing the uptake of vitamin C and its conversion to d-xylulose 5-phosphate and CO_2_^20^. UlaA, UlaB and UlaC are the components of the l-ascorbate PTS EII complex^21^ (Fig. 1a), involve in the uptake of vitamin C and its phosphorylation to l-ascorbate 6-phosphate^8^. UlaA from *Escherichia coli* in both outward-open and occluded conformations and homologs of soluble UlaB (PtxB; PDBID: 3CZC) and UlaC (PtxA; PDBID: 3BJV) from *Streptococcus mutans* have been solved by X-ray crystallography^11,22^ (Fig. 1a). Availability of the inward-facing structure of UlaA would facilitate a better understanding of the elevator mechanism of UlaA-mediated vitamin C transport, completing the transport cycle in the greater PTS-AG superfamily. To obtain the inward-facing conformation, we screened different possible mis-sense mutations of UlaA from *Escherichia coli* (ecUlaA) (e.g., G58A, G286A, and G58A/G286A)^23^, or homologs from other bacteria^19^, e.g., *V. cholera*, *P. multocida*, and *M. pulmonis*, in which cytoplasmic UlaB is fused to the C-terminus of the UlaA domain (Fig. 1a). UlaA from *Pasteurella multocida* (pmUlaA) was finally crystalized after being proteolyzed with trypsin from the wild-type pmUlaAB protein, with a best diffraction of 3.35 Å (Supplementary Table S1).

## Results

### Structure of pmUlaA

pmUlaA shares 63% sequence identity with ecUlaA. Both proteins have a similar number of residues, with pmUlaA containing a 5-aa insertion (after 413) and a single residue deletion at position 433 (Supplementary Figure S1). The protein crystallized as a homodimer in the asymmetric unit, and the structure was solved using molecular replacement with the ecUlaA’s core domain (PDB ID: 4PR9^11^) as the initial model (Supplementary Figure S2). The final model for one protomer contained residues 37 to 460 as well as a molecule of vitamin C. According to the high sequence similarity between pmUlaA and ecUlaA, a nomenclature has been adopted from ecUlaA for the structural components with 11 TMs (although the closely related protein VpeC (TC# 4.A.7.1.2) apparently lacks the 11th TM), four hairpin structures (HP1–4) and three horizontal amphipathic helix segments (AH1–3; Supplementary Figure S1). The HP motifs that contribute β-strands are important for vitamin C coordination^11^. However, possibly due to flexibility, AH1, TM1, AH2, AH3 are not visible in the crystal structure of pmUlaA (Fig. 1b, c and Supplementary Figure S1). As for ecUlaA, each protomer of pmUlaA, excluding TM11, contains an internal ‘inverted’ structural repeat. The first two helices in each repeat TM2 and TM6-7 gave rise to V-shaped motifs (V motif 1 and V motif 2), both of which interlocked to form the V-motif domain. The rest of the TMs and HPs in each repeat formed the transporter cores (core 1 and core 2). These two transporter-core subdomains, together with TM11, form the core domain of pmUlaA. The substrate vimtain C in the structure of pmUlaA is located close to the membrane surface on the cytoplasmic side (Fig. 1b); therefore this structure is identified as the inward-facing conformation. The dimerization interface between the two protomers of the pmUlaA homodimer is formed with the V motif domains, which is a large and mostly hydrophobic interface, with a buried surface area of ~894.8 Å^2^.

### Conformational changes between inward and outward structures

**Fig. 2:**
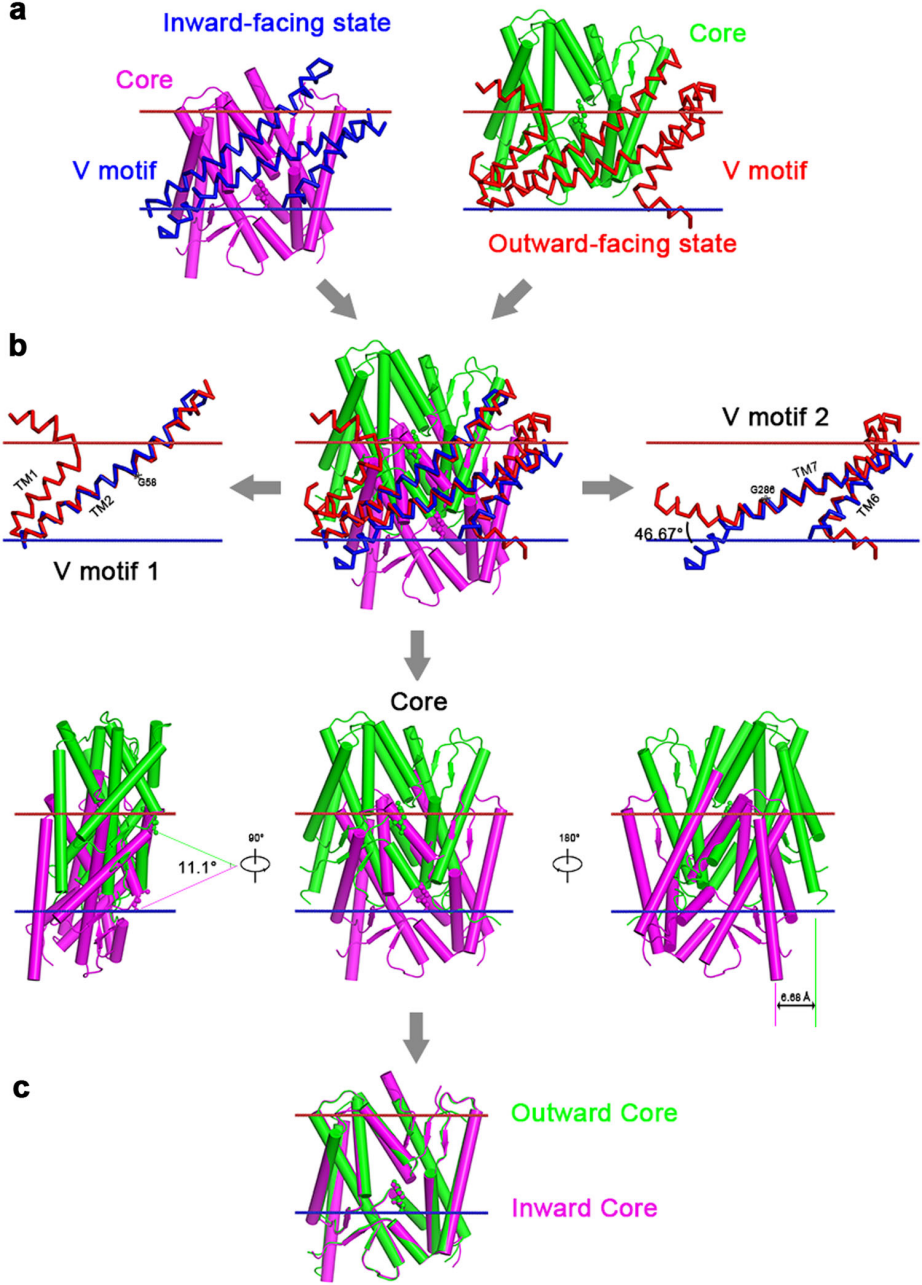
Comparison of inward-facing pmUlaA and outward-facing ecUlaA structures. **a** Orientations of pmUlaA and ecUlaA proteins in membranes as calculated with the PPM server. **b** Superimposition of pmUlaA and ecUlaA according to the transmembrane region of the V motif. TM2s of V motif 1 showed a small swing beyond the previously identified pivotal Gly58 position, while the C terminus of TM7 beyond Gly286 rotated about 46.67°. The whole core domain between pmUlaA and ecUlaA displayed a large rigid-body transformation, which includes an 11.1° rotation and a 6.68 Å translocation along an axis in the membrane. **c** Superposition of the core domains of pmUlaA and ecUlaA molecules

The inward-facing pmUlaA and outward-open ecUlaA structures align poorly when superposed over the entire monomer. However, when the two structures are superposed according to their orientation in membranes as predicted by the PPM server^24^ (Fig. 2a), the transmembrane parts of the V motifs align very well (Fig. 2b upper middle). The difference between the two structures can be described as the rearrangement of the cores and V motifs. The core domain undergoes a rigid-body rotation of 11.1° (Fig. 2b lower left) and a translation of 6.68 Å (Fig. 2b lower right) while transitioning from the outward-open to the inward-facing conformation with the substrate-binding site unchanged (Fig. 2c). While the ascorbate group translocator UlaA and secondary active transporters both contain an internal repeat, V motif domains, and core domains, UlaA lacks the third arm TM of each repeat which encases the core domain to transduce the stress when the core domain moves^25^. In the previous report, by comparing the two V-motif subdomains, “V-motif 1” and “V-motif 2” in each repeat, we proposed that the C-terminus of TM2 and TM7 possibly rotated to release the stress accomplished with core movement^23^. According to the sequence alignment of V-motif 1 and V-motif 2, we identified the highly conserved Gly58 and Gly286 in TM2 and TM7, respectively, as the potential pivots which are confirmed by the *in vitro* proteolipsome assays^23^. With the availability of inward-facing and outward-open structures, the conformational transitions of the C-terminus of TM2/TM7 become clear: the C-terminus of TM2 in V motif 1 undergoes minor changes (Fig. 2b upper left); however, TM7 in V motif 2 swings about 46.67° (Fig. 2b upper right). The other TMs remain stationary during the transport cycle.

### Active site architecture

In the ecUlaA structure, vitamin C was caged in an ellipsoid-shaped substrate-bound pocket of the core domain^11^. Thr86, Tyr87 from HP1, Asp314 from HP3, His194, Gln195 from HP2, Met410 from HP4 (corresponds to Met415 in pmUlaA because of the 5 residue insertion before HP4b), Gln139, Ile136, His135 from TM4a, Ile358, Phe362 from TM9a contribute a mixture of hydrogen bonds and van der Waals interaction with vitamin C (Supplementary Figure S3). Mutation of these active site binding residues to alanine or serine would cripple or abrogate the binding affinity for the substrate, as measured by isothermal titration calorimetry (ITC)^11^. The core domain of pmUlaA has the same binding residues as ecUlaA, except the threonine at 86th position is mutated to proline (Supplementary Figure S3). Once the Thr86 of ecUlaA is mutated to proline, one hydrogen bond from the hydroxyl group of threonine would be lost with the expectation of lesser binding affinity for the substrate—vitamin C. The binding affinity between ecUlaA-T86P and vitamin C was approximately 26.48 ± 6.1 μM (Supplementary Figure S4a), compared with the wild type ecUlaA of 6.1 ± 0.9 μM.

**Fig. 3:**
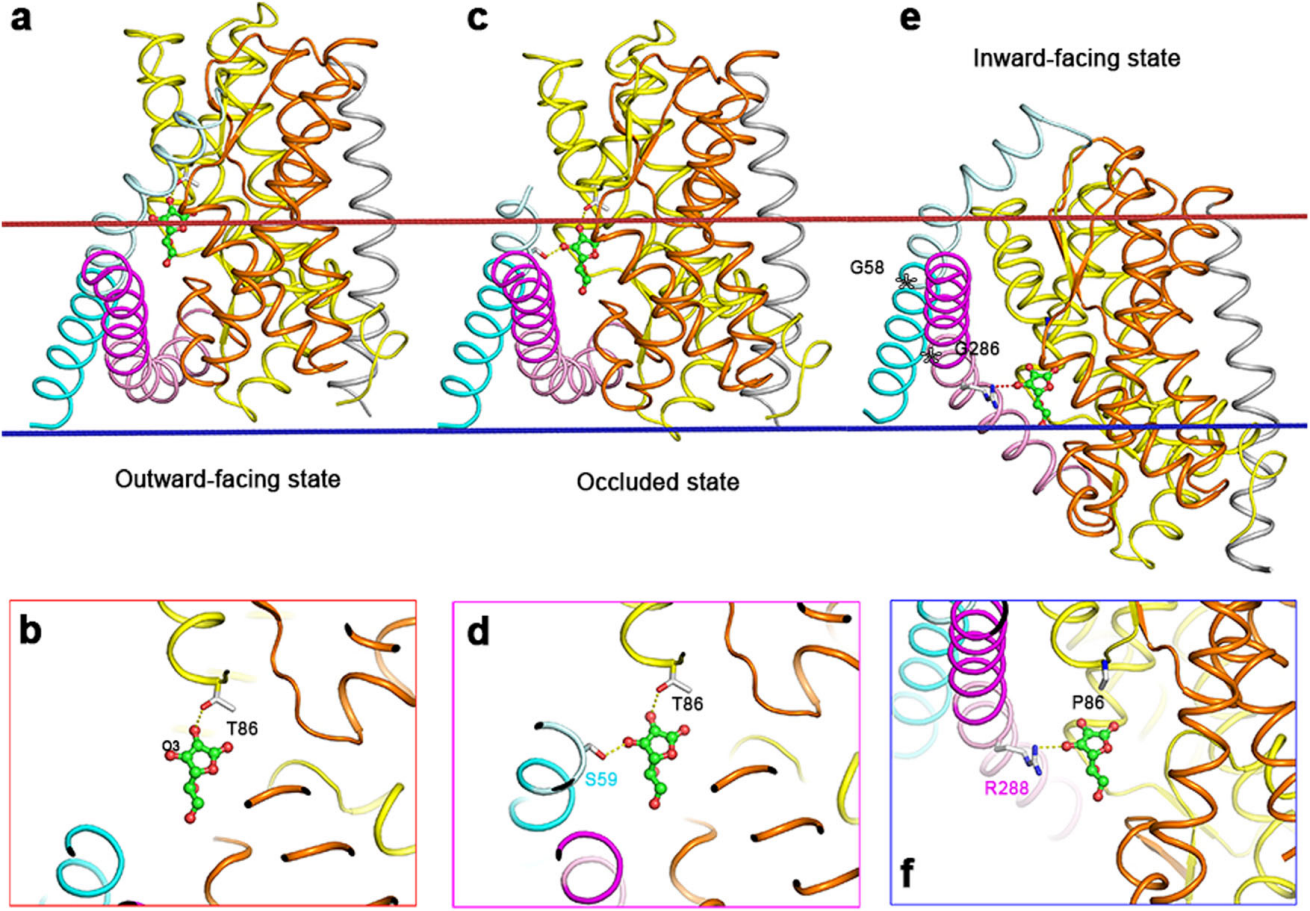
Vitamin C coordination. For clarity, only the substrate-coordinating residues different between pmUlaA and ecUlaA are shown here. **a**, **b** Substrate coordination in the outward-facing structure of ecUlaA. Vitamin C is entirely coordinated by the residues from the core domain. **c**, **d** Substrate coordination in the occluded structure of ecUlaA. Ser59 from V motif 1 contributes a hydrogen bond to the O3 atom of vitamin C, supposed to stabilize the charge balance of the substrate. **e**, **f** Substrate coordination in the inward-facing structure of pmUlaA. Arg288 from V motif 2 forms a hydrogen bond with the O3 atom of vitamin C

In the outward-facing structure of ecUlaA, the substrate (vitamin C) is entirely coordinated by the residues from the core domain (Fig. 3a, b, Supplementary Figure S3b). However, when the core domain moves toward the cytoplasmic side of the membrane, the serine at the 59th position from V motif 1 contributes a hydrogen bond to the O3 atom of vitamin C, which possibly stabilizes the charge balance of the substrate (Fig. 3c, d). As the substrate passes through the middle of the membrane, an arginine (R288) from V motif 2 forms a hydrogen bond with the O3 atom of vitamin C (Fig. 3e, f).

### Functional characterization of UlaA mutants with ecUlaA as a model system

**Fig. 4:**
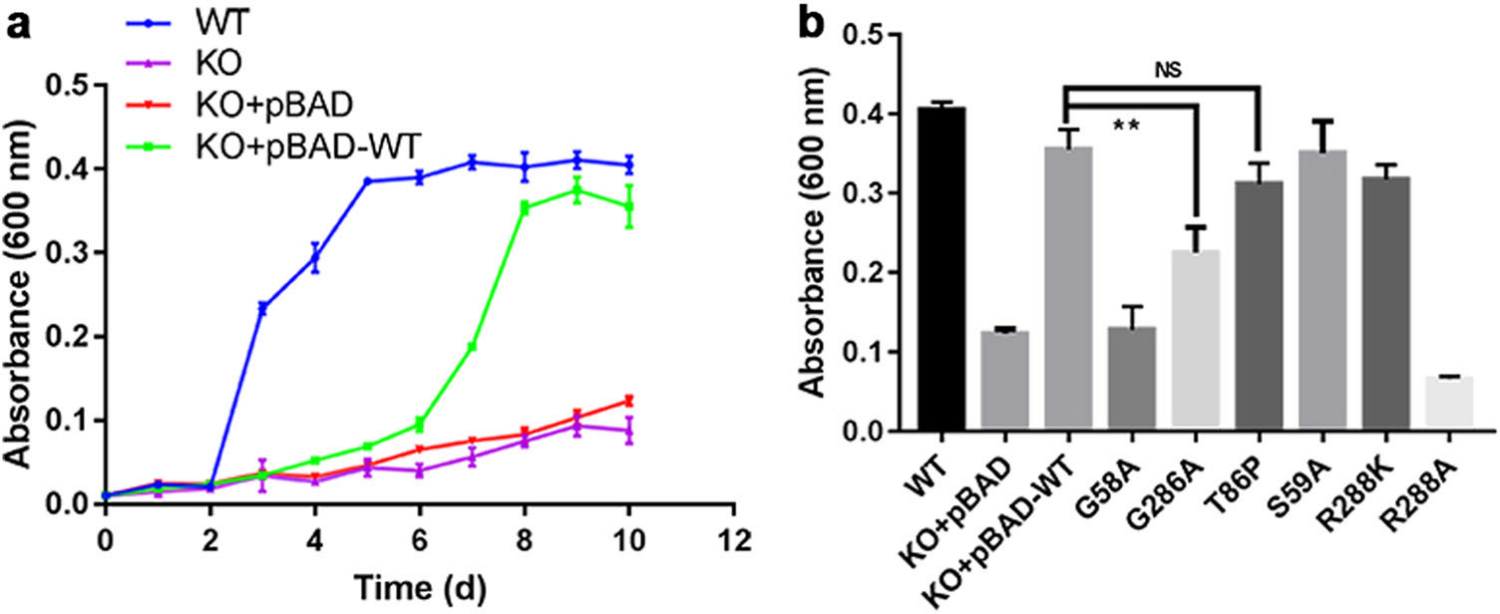
Growth studies. **a** Growth of *E. coli* strains on l-ascorbate. Growth of wild-type and mutant strains were conducted in minimal medium M9 under anaerobic conditions: blue, wild type; magenta, UlaA knock out; red, pBAD-empty vector; green, pBAD-WT. **b** Growth assay of several ecUlaA variants. Each experiment was repeated three times. Values are mean ± s.e.m. NS, not significant; **p < 0.05

To corroborate structural observations, we set up a growth assay to monitor the growth rate and growth extent of *E. coli* cells under anaerobic condition with l-ascorbate as the sole source of carbon and energy. In-frame deletion in the coding region of ecUlaA resulted in complete loss of l-ascorbate utilization (Fig. 4a). The presence of the pBAD-WT plasmid restored anaerobic growth on l-ascorbate to wild-type yields at 10 days. Replacement of pivotal residues on V motif domain Gly58 or Gly286 by Alanine invariably diminished l-ascorbate uptake, therefore cell growth, which is consistent with our in vitro proteoliposome assays^23^. By contrast, the ecUlaA variant Thr86Pro exemplified in pmUlaA showed no significant reduction of growth (Fig. 4b). Replacement of Ser59 from V motif 1 by Ala has no effect on l-ascorbate utilization. Changing Arg288 from V motif 2 to the Lys variant maintains the hydrogen bond between the O3 atom of the substrate and the N^ω^ atom of the residue, which neither changes the electron charges of the binding surface of UlaB, nor results in any effect on the transport of l-ascorbate. However, the Arg288Ala variant abrogated the l-ascorbate utilization completely, possibly because the interaction interface between UlaA and UlaB was altered, which resulted in the abolishment of phosphate group transfer from UlaB to the UlaA-bound substrate.

## Discussion

### Mechanism of membrane transport

**Fig. 5:**
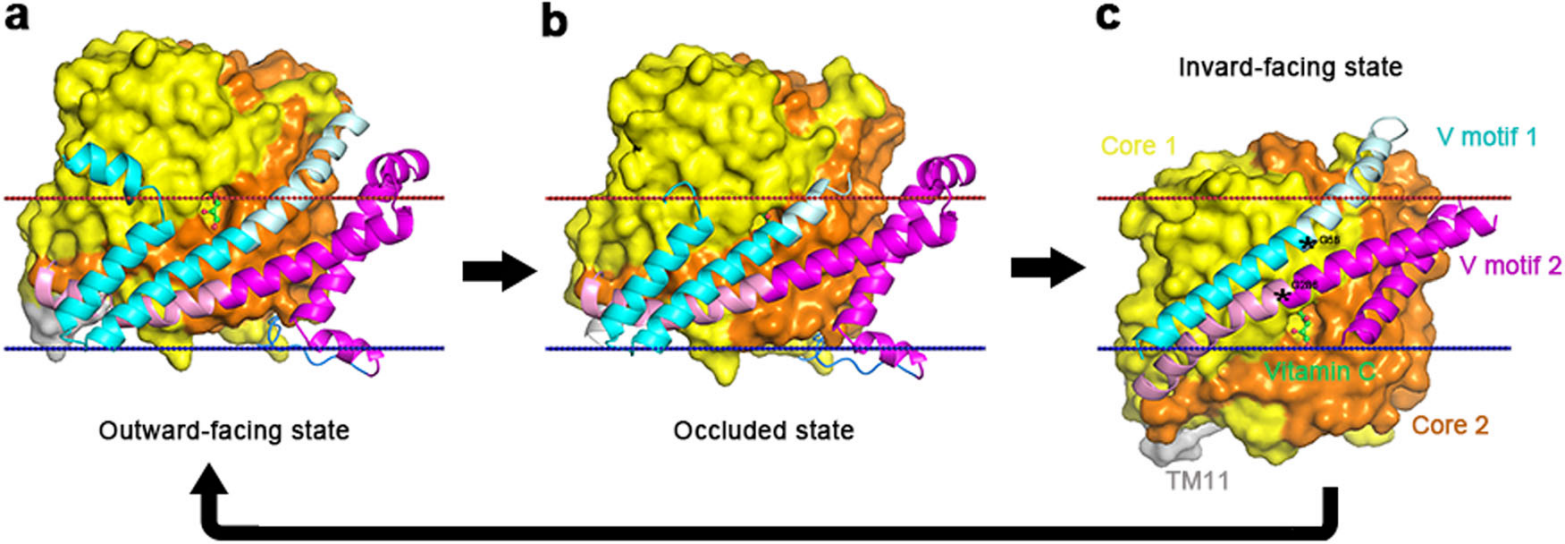
Working model for the transport mechanism of UlaA. Outward-facing, occluded, and inward-facing structures of UlaA revealed the complete transport cycle of EIIC of the PTS-AG family. The stationary part of V motif 1 is shown in cyan; the swing part of V motif 1 in palecyan, core 1 in yellow, the stationary part of V motif 2 in magenta, the swing part of V motif 2 in pink, the core 2 in orange and TM11 in gray. Vitamin C is shown in ball-and-stick representation

Topologically, UlaA more closely resembles secondary carriers^26^, with the elevator mechanism, another form of the alternating access mechanism, for substrate transport, especially similar to that of Glt_Ph_^25,27^. However, due to the lack of the third arm helix in each repeat as secondary carriers, the V motif subdomain is separated into two functional parts: membrane-spanning parts TM1/TM2 N-terminus and TM6/TM7 N-terminus, and pivot-swinging parts TM2 C-terminus and TM7 C-terminus. The transport activity of UlaA may involve four sequential steps (Fig. 5). The default state is likely to be in an outward open state, similar to the ecUlaA outward-open structure without the bound substrate (not shown). In this state, “Core 2” subdomain (orange) approaches the “V-motif 2” subdomain (magenta) (Fig. 5a). Then, substrate binding to the pocket of the core domain leads to a switch to an inward-facing state, through the movement of the Core relative to the V-motif. In this inward-facing state (pmUlaA), “Core 1” (yellow) is close to “V-motif 1” subdomain (cyan), and substrate pocket is accessible from the cytoplasmic side (Fig. 5c). In the fourth step, UlaB transfers the phosphate anion to vitamin C coupled with energy^19,20^ (Supplementary Figure S5). As a result, the resultant product l-ascorbate-6-P might leave the binding site and enter into the cytosol. Finally, utilizing the energy coupled with the transferred phosphate originally from PEP, the core domain returns back to the default state and the whole system will restart this novel cycle of transport. Based on the structural analysis and probable rotational freedom of degree of position with the residue glycine, the C-terminus of the last TM in two V-motif subdomains will rotate, pivoted on highly conserved Gly58 and Gly286, to come along with the movement of the core domain.

Our inward-facing UlaA structure completes the transport cycle of PTS-AG family. Based on our structural observations, the proposed mechanism of molecular motion is consistent with the previous work of the glutamate transporter homolog Glt_Ph_^27^. And the structural feature of intermolecular pseudo-symmetry in UlaA is similar as the other secondary transporters^25^ (e.g., LeuT^28^, AdiC^29–31^, NpaA^16^).

## Materials and methods

### Protein expression and purification

We screened multiple UlaA homologs from different prokaryotic species in order to obtain an inward-facing conformation structure. Those genes were cloned into pET21b (Novagen) with a C-terminal His x 8 tag. The transformed C43 (DE3) (Lucigen) cells were grown in Luria broth at 37 ℃ and induced with 0.2 mM isopropyl-β-d-thiogalactopyranoside (IPTG) after the OD_600_ reached 1.2. Cells were disrupted with a French Press with two passes at 15,000 p.s.i., in buffer A containing 25 mM Tris-Cl, pH 8.0, and 150 mM NaCl. After a low-speed centrifugation, the resulting supernatant was centrifuged at high speed to sediment a membrane fraction, which then was incubated in buffer A with 1% (w/v) n-dodecyl β-d-maltopyranoside (DDM, Anatrace) and 2 mM ascorbic acid (Sigma-Aldrich) for 1 h at 4 °C. The lysate was centrifuged again, and the supernatant was loaded onto a Ni^2+^-NTA affinity column (Qiagen). After three washes, the protein was eluted with 25 mM Tris-Cl, pH 8.0, 150 mM NaCl, 250 mM imidazole, 2 mM ascorbic acid and 0.56% (w/v) n-nonyl- β-D-maltopyranoside (NM, Anatrace), and it was then concentrated by Amicon Ultra (Millipore) for subsequent gel filtration in buffer A with detergent and 2 mM ascorbic acid. The UlaA domain from *Pasteurella multocida* showed a high yield and good behavior in gel filtration. The peak fractions were collected for crystallization.

### Crystallization

Crystals were grown after 4 days at 18 °C by the hanging-drop vapor-diffusion method. The *Pasteurella multocida* UlaA domain (1-465) protein purified in 0.2% (w/v) n-decyl-β-d-maltopyranoside (DM; Anatrace) gave rise to large plate-shaped crystals in 0.28 M CaCl_2_, 0.1 M Tris-HCl, pH 7.5,42% PEG400 conditions. The best data set collected at a synchrotron for these crystals, at a resolution of 6.5 Å, showed an inward-facing conformation. A detergent screen found 3-cyclohexyl-1-propylphosphocholine (Cyclofos-3, Hampton Research) at 43 mM, final concentration, improved the diffraction to 4.5 Å. Purified protein in 0.4% (w/v) n-nonyl-β-d-glucopyranoside (β-NG, Anatrace) with 43 mM Cyclofos-3 gave rise to cubic-shaped crystals. To further improve the resolution, 0.01 M betaine hydrochloride (Sigma-Aldrich) was added into crystallization buffer to improve diffraction to 3.35 Å. All crystals were directly flash frozen in a cold nitrogen stream at 100 K.

### X-ray data collection and structure determination

Native X-ray data at 3.35 Å resolution was collected on SSRF beamline BL17U1. The data were integrated and scaled using HKL2000^32^. Further processing was carried out using programs from the CCP4 suite^33^.

The core domain of ecUlaA (PDB ID: 4RP9^11^) was used as the model for molecular replacement with PHASER^34^. One distinct solution was identified. The resulting electron density was of sufficiently good quality, and most of the side chains were clearly shown. Additional missing residues were added in COOT^35^ manually. The structure was refined with PHENIX^36^. Model validation was performed with PROCHECK^37^ and the WHATCHECK routine of WHAT IF^38^. All structure figures were prepared with PyMOL^39^.

### Generation of a UlaA-disrupted E. coli strain

A gene-disrupted *E. coli* strain was generated by the methods described by Datsenko and Wanner^40^. BW25113 cells carrying the Red helper plasmid pKD46 were cultured at 30 °C in SOB medium containing ampicillin and 1 mM l-arabinose, and electroporation competent cells were prepared with ice-cold 10% glycerol when the OD_600_ reached 0.4. The FRT-flanked kanamycin resistance sequence in the pKD4 plasmid was amplified using the primers UlaA-F/UlaA-R. The PCR product was gel-purified, treated with DpnI and re-purified. 1 μg DNA was transformed into 50 μl of competent cell by electroporation with Pulse Controller at 2.5 kV with 25 mF and 200 Ω in chilled 0.2 cm electroporation cuvettes (Bio-Rad), followed by the immediate addition of 1 ml SOC medium containing 10 mM l-arabinose. After incubation at 37 °C for 2 h, the culture was spread out onto a kanamycin-containing LB-agar plate and incubated at 37 °C overnight. To verify that the UlaA sequence was replaced by the FRT-flanked kanamycin resistance sequence, primers UF/DR, UF/Km-R, Km-F/DR were used to amplify the chromosome DNA from the Km-resistance transformants. UF and DR were both the neighboring gene-specific primers; the Km-R and Km-F were Km gene-specific primers.

To eliminate the Km gene in the chromosome, the pCP20 plasmid, which expresses the FLP recombinase, was transformed into Km-resistance cells and selected for ampicillin resistance on an LB-agar plate at 30 °C. The tranformants were incubated at 43 °C overnight and then placed onto an LB-agar plate. Several independent colonies were selected and tested by PCR using primers UF/DR. PCR products were analyzed by both DNA electrophoresis and DNA sequencing. All primers used for gene knock-out experiment were listed in Supplementary Table S2.

### Growth assay

Wild type or mutated UlaA coding DNA were subcloned into the pBAD24 vector individually and transformed into UlaA-disrupted BW25113 cells. To measure cell growth under the condition that l-ascorbic acid was used as the sole carbon source, cells were grown anaerobically in M9 minimal medium plus 20 mM l-ascorbic acid (M9A medium), concentration of the L-ascorbic acid was tested to be best for *E. coli* growth^19^. Briefly, Cells grown in LB medium were collected, adjusted to OD_600_ = 1.0 with M9 medium, and then inoculated 1:100 into screw-cap culture tubes. Ampicillin and 2 mM l-arabinose were added into the medium when pBAD24 were used as the expressing plasmid. These tubes were filled to the top with M9A medium, sealed with parafilm, and then incubated at 37 °C without agitation. The OD_600_ values were measured at 24 h intervals.

### Isothermal titration calorimetry (ITC)

The assays were performed as described^11^.

### Accession code

The structural coordinate and structural factors have been deposited into the Protein Data Bank (PDB) with code of 5ZOV.

## Electronic supplementary material

Supplemental material

Movie of L-ascorabate transportation across the cell membrane by UlaA
