# Supplementary material for: Inward-facing conformation of l-ascorbate transporter suggests an elevator mechanism
Source: Cell Discov. 2018 Jul 17;4:35. doi: 10.1038/s41421-018-0037-y (PMC6048161; doi:10.1038/s41421-018-0037-y)
Supplement: Supplementary file 1 — Supplemental material [file 41421_2018_37_MOESM1_ESM.doc]

**Supplementary Table S1. Statistics of data collection and refinement**.

| Data | pmUlaA (inward-facing) |
| --- | --- |
| Space Group | P3_2_ |
| Unit Cell (Å,°) | a = b = 107.32 Å, c = 113.27 Å, γ = 120° |
| Number of molecules in ASU | 2 |
| Wavelength (Å) | 0.97915 |
| Resolution (Å) | 50~3.35 (3.47~3.35) |
| R_merge_ (%) | 5.9 (84.1) |
| I/sigI | 23.0 (1.87) |
| Completeness (%) | 97.6 (100) |
| Total number of reflections | 88,156 |
| Unique reflections | 20,489 |
| Redundancy | 4.3 (4.7) |
| Wilson B factor (Å^2^) | 58.35 |
| R-factor / R_free_ (%) | 27.32 / 31.65 |
| Average B value (Å^2^) / No. of atoms |  |
| Overall | 89.58 / 6164 |
| Main chain | 89.22 / 3192 |
| Side chain | 90.29 / 2946 |
| Other entities | 54.82 / 26 |
| R.M.S. deviations from ideal values |  |
| Bonds (Å) | 0.005 |
| Angle (°) | 1.001 |
| Ramachandran plot statistics (%) |  |
| Most favorable | 89.5 |
| Additionally allowed | 8.6 |
| Generously allowed | 1.3 |
| Disallowed | 0.6 |

Values in parentheses are for the highest resolution shell. *R_merge_*=Σ_h_Σ_i_|*I_h,i_*-*I_h_*|/Σ_h_Σ_i_*I_h,i_*, where *I_h_* is the mean intensity of the *i* observations of symmetry related reflections of *h*. *R*=Σ|*F_obs_*-*F_calc_*|/Σ*F_obs_*, where *F_calc_* is the calculated protein structure factor from the atomic model (*R*_free_ was calculated with 5% of the reflections selected randomly).

**Supplementary** Table S2 Primers used in the gene knock-out experiment

| Primer | Sequence(5’-3’) |
| --- | --- |
| UlaA-F | ACGCCAGCCGCCGAAGTGGTTAGCGAACACGCCGATGGTGGCGTTCATATGAATATCCTCCTTAG |
| UlaA-R | CCGGTGGTGGCGAAAATGTCCGAAGTCTACGGCATTAACGGCGCAGTGTAGGCTGGAGCTGCTTC |
| UF | GTGCCAGGAGCGACCTGTTCTTGTTCAATTT |
| DR | AGCCTGCAGGCGGATGGATTTATTTTCCGC |
| Km-R | GTCGTGGCCAGCCACGATA |
| Km-F | GTGGAAAATGGCCGCTTTTC |


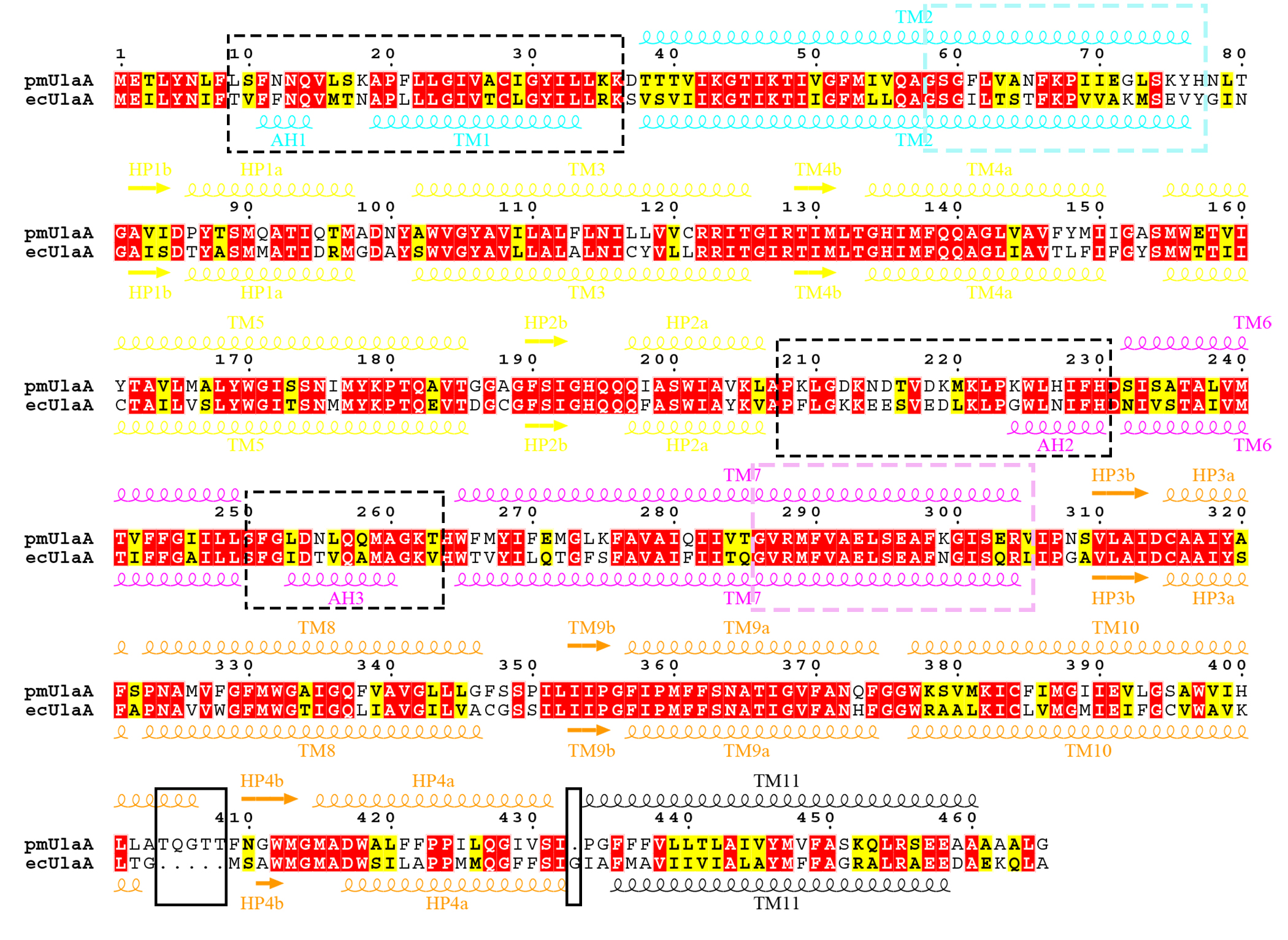


**Supplementary Figure S1 Sequence alignment of the *ec*UlaA and pmUlaA.** Secondary structural elements of pmUlaA and ecUlaA are indicated above and below the sequence alignment. Conserved amino acids are colored red and yellow in decreasing degrees of conservation. UlaA is spatially organized into “V motif 1” (cyan), “Core 1” (yellow), “V motif 2” (Magenta), “Core 2” (orange), and “TM11” (gray) subdomains. The secondary structures missing in the pmUlaA crystal structure are labelled with the dashed black square. Black squares indicate the insertion or deletion in pmUlaA. Cyan and magenta dashed squares correspond to the swing part of the V motif after the pivotal position Gly58/Gly286.


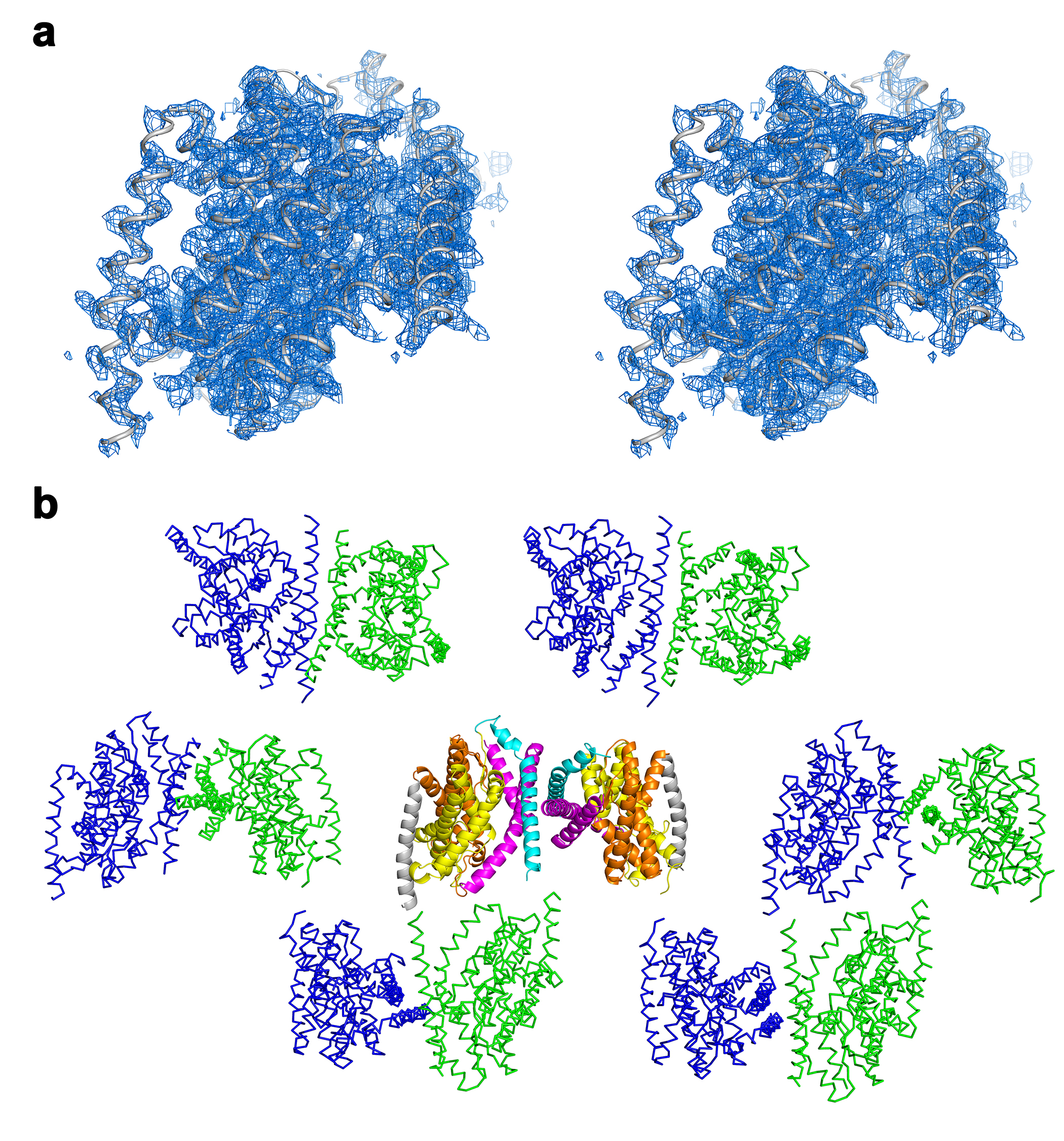


**Supplementary Figure S2 Structure determination of pmUlaA. a.** *2Fo-Fc* electron density map contoured at 1.5 σ of one pmUlaA protomer at the stereo view. **b.** Crystal packing of pmUlaA molecule is shown, with a homodimer in the asymmetric unit as cartoon representations.

**
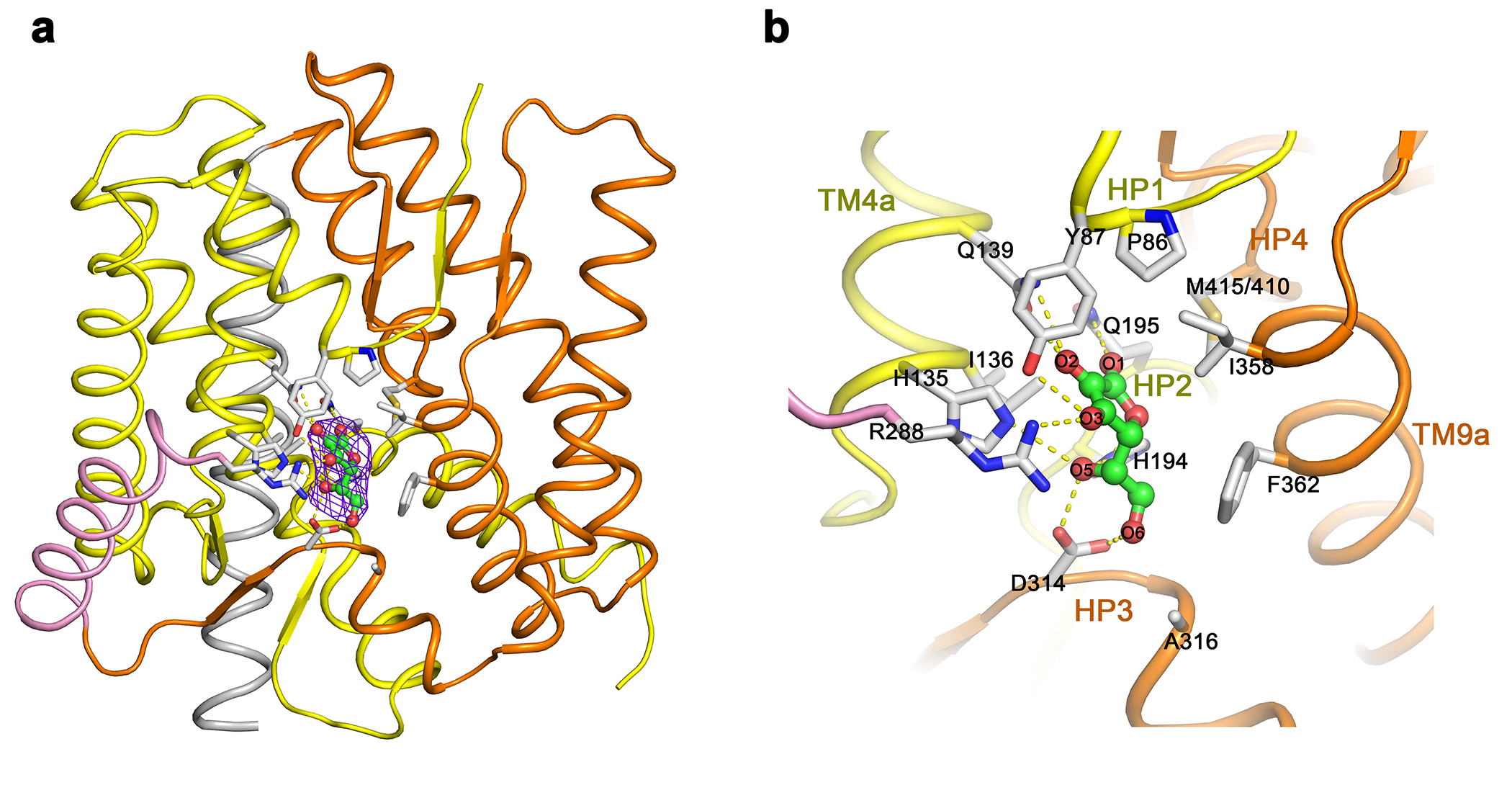
**

**Supplementary Figure S3 Vitamin C coordination. a.** SA-omit map of vitamin C contoured at 2 σ. **b.** Polar and van der Waals contacts coordinating vitamin C. Hydrogen bonds are indicated with the yellow dashed lines.

**
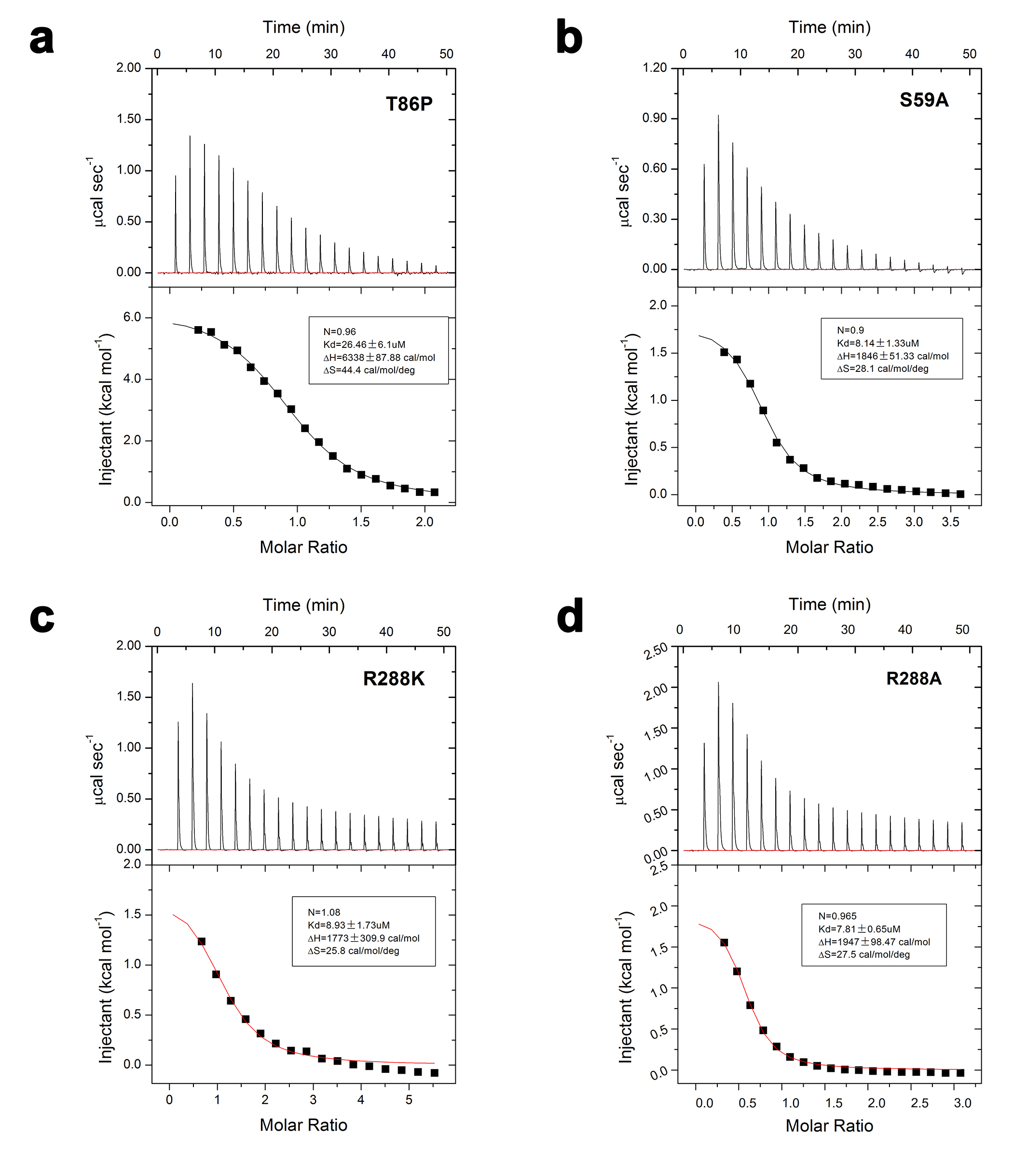
**

**Supplementary Figure S4 The mis-sense mutations in ecUlaA.** Measurement of binding affinity between L-ascorbate and the ecUlaA-T86P (a), S59A (b), R288K (c), R288A (c) proteins by isothermal titration calorimetry (ITC). The experiment was carried out as described[^1^](#_ENREF_1).

**
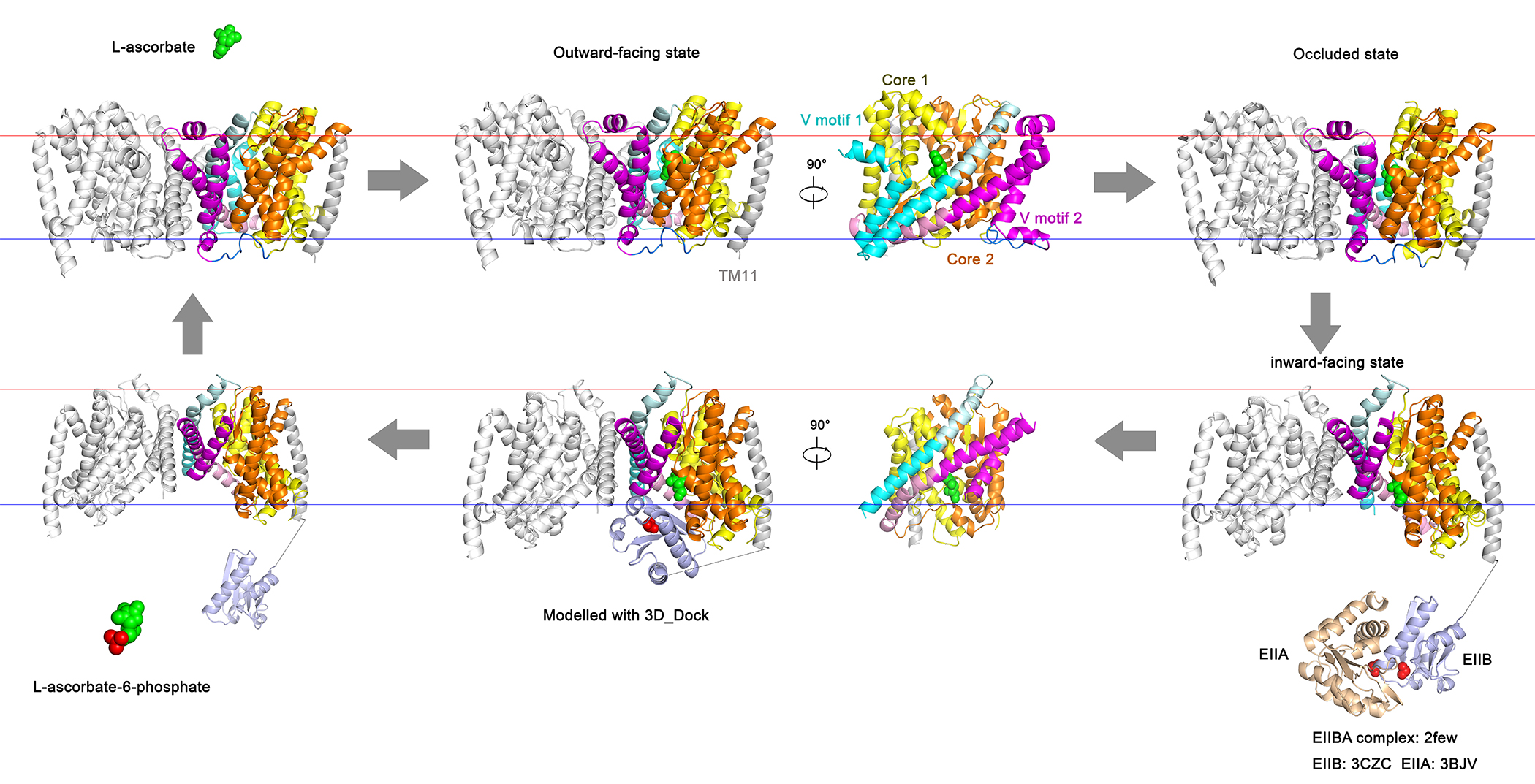
**

**Supplementary Figure S5 Working model for the transport mechanism of PTS EII complex.** The transport activity of UlaA may involve four sequential steps. The default state is likely to be in an outward open state, similar to the ecUlaA outward-open structure without the bound substrate (upper left). In this state, “Core 2” subdomain (orange) approaches “V-motif 2” subdomain (magenta). Then, substrate binding to the pocket of the core domain leads to switch to an inward-facing state, through the movement of Core relative to V-motif. In this inward-facing state (lower right), “Core 1” (yellow) is close to “V-motif 1” subdomain (cyan), and substrate pocket is accessible from cytoplasmic side. In the fourth step, UlaB transfers the phosphate anion obtained from UlaC to vitamin C coupled with energy. As a result, the resultant product L-ascorbate-6-P might leave away from the binding site and entry into the cytosol (lower left). Finally, utilizing the energy coupled with the transferred phosphate originally from PEP, the Core domain returns back to the default state and the whole system will restart a novel cycle of transportation. UlaB and UlaC were modelled with the corresponding structures from *Streptococcus mutans* (PDB IDs: 3CZC & 3BJV[^2^](#_ENREF_2)) by the homologue modelling method. The complex between EIIA and EIIB were modelled according to the NMR structure of EIIA^mtl^ and EIIB^mtl^ (PDB ID: 2FEW[^3^](#_ENREF_3)). UlaB and inward-facing UlaA complex was docked with 3D_dock program[^4^](#_ENREF_4).

**References**

1 Luo, P. *et al.* Crystal structure of a phosphorylation-coupled vitamin C transporter. *Nature structural & molecular biology* **22**, 238-241, doi:10.1038/nsmb.2975 (2015).

2 Lei, J., Li, L. F. & Su, X. D. Crystal structures of phosphotransferase system enzymes PtxB (IIB(Asc)) and PtxA (IIA(Asc)) from Streptococcus mutans. *J Mol Biol* **386**, 465-475, doi:10.1016/j.jmb.2008.12.046 (2009).

3 Suh, J. Y., Cai, M., Williams, D. C., Jr. & Clore, G. M. Solution structure of a post-transition state analog of the phosphotransfer reaction between the A and B cytoplasmic domains of the mannitol transporter IIMannitol of the Escherichia coli phosphotransferase system. *The Journal of biological chemistry* **281**, 8939-8949, doi:10.1074/jbc.M513466200 (2006).

4 Gabb, H. A., Jackson, R. M. & Sternberg, M. J. Modelling protein docking using shape complementarity, electrostatics and biochemical information. *J Mol Biol* **272**, 106-120, doi:10.1006/jmbi.1997.1203 (1997).
